# All-optical attosecond time domain interferometry

**DOI:** 10.1093/nsr/nwaa211

**Published:** 2020-09-04

**Authors:** Zhen Yang, Wei Cao, Yunlong Mo, Huiyao Xu, Kang Mi, Pengfei Lan, Qingbin Zhang, Peixiang Lu

**Affiliations:** Wuhan National Laboratory for Optoelectronics and School of Physics, Huazhong University of Science and Technology, Wuhan 430074, China; Wuhan National Laboratory for Optoelectronics and School of Physics, Huazhong University of Science and Technology, Wuhan 430074, China; Wuhan National Laboratory for Optoelectronics and School of Physics, Huazhong University of Science and Technology, Wuhan 430074, China; Wuhan National Laboratory for Optoelectronics and School of Physics, Huazhong University of Science and Technology, Wuhan 430074, China; Wuhan National Laboratory for Optoelectronics and School of Physics, Huazhong University of Science and Technology, Wuhan 430074, China; Wuhan National Laboratory for Optoelectronics and School of Physics, Huazhong University of Science and Technology, Wuhan 430074, China; Wuhan National Laboratory for Optoelectronics and School of Physics, Huazhong University of Science and Technology, Wuhan 430074, China; Wuhan National Laboratory for Optoelectronics and School of Physics, Huazhong University of Science and Technology, Wuhan 430074, China; Hubei Key Laboratory of Optical Information and Pattern Recognition, Wuhan Institute of Technology, Wuhan 430205, China; CAS Center for Excellence in Ultra-intense Laser Science, Shanghai 201800, China

**Keywords:** interferometry, attosecond, all-optical, precision measurement

## Abstract

Interferometry, a key technique in modern precision measurements, has been used for length measurement in engineering metrology and astronomy. An analogous time-domain interferometric technique would represent a significant complement to spatial domain applications and require the manipulation of interference on extreme time and energy scales. Here, we report an all-optical interferometer using laser-driven high order harmonics as attosecond temporal slits. By controlling the phase of the temporal slits with an external field, a time domain interferometer that preserves both attosecond temporal resolution and hundreds of meV energy resolution is implemented. We apply this exceptional temporal resolution to reconstruct the waveform of an arbitrarily polarized optical pulse, and utilize the provided energy resolution to interrogate the abnormal character of the transition dipole near the Cooper minimum in argon. This novel attosecond interferometry paves the way for high precision measurements in the time-energy domain using all-optical approaches.

## Teaser

An all-optical time domain Young's interferometer using attosecond pulses is implemented, and has been successfully used for precision measurement in time-energy domain.

## INTRODUCTION

Young's double slit and equivalent two-arm interference experiments, as direct proof of the wave nature of light and matter, have been used to test the basic principles of quantum mechanics [1–4]. Apart from the pivotal role they have played in understanding the quantum world, such interferometric schemes are also sensitive tools for applications in high precision measurement. For example, photoionization from molecules is equivalent to an atomic scale interferometer [5–7], which offers superb spatial resolution for accessing chemical bond length [6,7]. A more versatile approach for interferometer-based precision measurement changes the phase of individual arms by inserting targets to be investigated. In this approach, the wave-front of the incoming waves at the slits is effectively manipulated, leading to profound changes in the final interference pattern from which quantities related to the inserted objectives can be accurately extracted.

Similar wave-front manipulation schemes have been employed to extend precision measurements to exceedingly fast processes in the time-domain [8–10]. Recently, an all-optical interferometer combining isolated attosecond pulses with Young's configuration has been developed. Two spatially separated, extreme ultraviolet (EUV) isolated attosecond pulses interfere in the far field. By introducing a weak optical pulse to perturb one arm, the angular distribution of the far field interference pattern acquires a displacement used to map the waveform of the vectorial optical field [10]. This all-optical interferometric scheme provides a robust and reliable means for the direct sampling of the electric field of an optical pulse with high precision, serving as an important complementary technique to existing schemes based on attosecond streaking [11,12] and avoiding distortion issues encountered in non-interferometric sampling approaches based on perturbed high-harmonic generation (HHG) [13,14]. Attosecond two-source interferometers have also been introduced by using two foci [15], mixed gases [16] or separated targets [17] to generate two independent HHG beams. By delaying the two attosecond beams relative to each other, accurate phase information associated with atomic transitions is encoded in interference fringes. The aforementioned interferometers possess either high temporal resolution for waveform characterization or sufficient frequency resolution for recovering phase information.

Developing an interferometric scheme that achieves high temporal and spectral resolution simultaneously is essential for enhancing our understanding of light–matter interactions. The contradiction between high temporal and spectral resolution may be circumvented with a pulse train. As demonstrated by M Isinger *et al.* [18], laser-assisted photoemission of neon using an attosecond pulse train creates a photoelectron spectrogram bearing high temporal and adequate spectral resolution simultaneously. Different photoionization pathways nearby in energy are disentangled, allowing for unambiguous determination of the photoemission time delay, thereby solving a long-standing puzzle [19–21]. Therefore, the development of all-optical interferometric techniques based on attosecond pulse trains promises to extend high precision measurements to the time-energy domain using simple and robust approaches. In this work, we present an all-optical time-domain few-slit interferometer that incorporates an attosecond pulse train. By manipulating the temporal wave-front of the pulse train in a controlled manner, we demonstrate that this interferometer combines both time and spectral resolution in a single measurement, permitting multiple high precision applications. In the first application, we successfully recover the waveform of a perturbing field and implement an all-optical petahertz oscilloscope. In a second application, the intrinsic energy resolution provided by the interferometer allows for the energy-resolved analysis of the dynamics of EUV generation. Subtle changes as small as a few tens of attoseconds in the attosecond pulse separation are trackable and attributed to the presence of a Cooper minimum in the generating medium.

## PRINCIPLE OF ATTOSECOND FEW-SLIT INTERFEROMETRY

**Figure 1.**
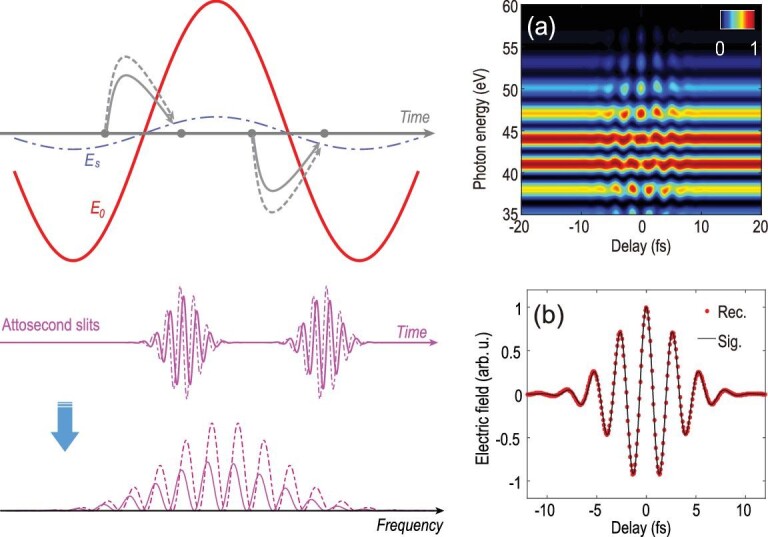
Principle of attosecond few-slit interferometry. Strong driving pulse $E_{\! 0}$ generates high order harmonics every half optical cycle of the driver, the attosecond bursts (pink solid lines) maintain mutual coherence and are equivalent to a Young's interferometer with the attoecond pulses as the slits. The interference leads to fringes in the frequency domain. When a weak signal pulse $E_{\!\! S}$ is synchronized with the driver, it perturbs the electron trajectories (grey curved arrows) for harmonic generation and imposes additional phase in each attosecond slit. This will induce a shift of the interference pattern in the frequency domain. (a) The simulated harmonic spectrum using strong field approximation indicates that the energy shift of the harmonics is sensitive to the relative delay between the driving field and signal pulse. The delay dependent energy shift of a single harmonic can be expressed as $\sigma (\tau) \propto E_{\!\! S}(\tau) + \alpha E_{\!\! S}(\tau + \Delta)$ (see the text), which can be used to directly reconstruct the electric field of the signal pulse. (b) The reconstruction of waveform of the signal using harmonic around 42 eV, the reconstructed (red dotted lines) and original (black solid lines) field agree with each other.

The time domain interferometer is based on laser-driven EUV radiation [22,23]. The principle of the method is illustrated in Fig. 1. When the phase matching condition is fulfilled, EUV radiation is launched within a few time windows with a duration of a few hundred attoseconds each near the peak of the driving field, producing an EUV pulse train [22,24]. Using this pulse train configuration, the interferometer is analogous to the conventional spatial double-slit experiment except that temporal slits are used for diffraction instead. This experimental concept has been successfully demonstrated in matter waves using nanosecond [25], femtosecond [26] and attosecond time slits [27]. As a result of interference between radiation from different time windows, discrete frequency components, known as high order harmonics, are created [28,29]. When a weak signal pulse is applied, it perturbs the harmonic generation process and introduces a phase variation in the wave-front of the time slits [13,30]. As a result, each high order harmonic experiences a prominent energy shift upon which temporal structure of both the perturbing signal and the attosecond slits is imprinted.

The harmonic energy shift can be derived within the framework of the strong field approximation (SFA) [31]. High order harmonics are emitted every half optical cycle of the driving field. For simplicity, we consider a double-slit interferometer configuration. The phase difference of harmonics radiating from two consecutive attosecond slits in absence of the perturbing signal is $\Delta \phi({\rm{\omega }} ) = \frac{{{\rm{\omega T}}}}{2}\ + \pi - \delta $, where T is the optical cycle of the driver. When the driving pulse is short, the electric field amplitude from one half cycle to the next will change, leading to a blue shift of the high harmonics. Such a non-adiabatic effect has been reported in [32] and is accounted for by $\delta $ in the expression above. When a signal pulse is present, it introduces an additional phase on each attosecond slit and the total phase difference is given by (see section I of the supplementary data):

$$\begin{eqnarray*}
\Delta {\rm{\Phi}}({{\rm{\omega}},{\rm{\tau}}}) &=& \frac{1}{{72}}\ \alpha {E_0}{\omega _0}t_d^4{E_s}\! ({\tau + \Delta})\\
+\, \frac{1}{{72}}{E_0}{\omega _0}t_d^4{E_s}\!(\tau) + \Delta \phi ({\rm{\omega}}),
\end{eqnarray*}$$

where *E_0_* and *E_s_* are the amplitudes of the electric field of the driver and signal, respectively; *t_d_* stands for the excursion time in the three-step model of high harmonic generation; $\tau $ is the delay between the signal and driving pulse; and ${\rm{\Delta }}$ is the time separation between the consecutive attosecond pulses. The coefficient $\alpha $ is introduced to account for the intensity variation of the driving field within an optical cycle and is close to 1 in general. The EUV spectral maxima are then determined by the constructive interference condition ${\rm{\Delta \Phi}}({{\rm{\omega}},{\rm{\ \tau }}} ) = \ 2m\pi $, with $m$ being an integer. Therefore, the harmonics will peak at:

(1) \begin{eqnarray*} \omega \ &=& ({2m - 1}){\omega _0} + \frac{{2\delta }}{T} - \frac{{{E_0}{\omega _0}t_d^4}}{{36T}}\nonumber\\ && \times\, \big({\alpha {E_s}\! ({\tau + \Delta}) + {E_s}\!(\tau} \big).\end{eqnarray*}

The first term in equation (1) indicates that odd harmonics are produced by a continuous laser. The second term corresponds to the non-adiabatic effect-induced blue shift of the harmonics. The last term is a quantitative description of the influence of the perturbing signal pulse. The signal pulse-induced energy shift is then:

(2) \begin{equation*} {\rm{\sigma}}\!({\rm{\tau}}) \propto {E_s}\!(\tau) + \alpha {E_s}\!({\tau + \Delta}).\end{equation*}

Equation (2) shows that the shift of the harmonic peak is proportional to a linear combination of two delayed signal pulses and describes the quantitative connection between the interference pattern and the electric field of the perturbing pulse. An immediate application of this interferogram is for real-time probing of the electric field of an optical pulse. Figure 1(a) shows the delay-dependent high harmonic spectrum calculated using the SFA [31]. As the two pulses are well separated in time, the signal has a negligible impact on the harmonic spectrum because the signal pulse is too weak to contribute to the harmonic yield alone. When the delay approaches the overlap region, delay-dependent oscillations in both the flux and central frequency of each harmonic arise. The profound energy shift of each harmonic in the overlap region is governed by equation (2). To precisely retrieve the perturbed electric field, the quantity $\alpha $ has to be determined. $\alpha $ depends on the waveform of the driving pulse and depicts the amplitude variation of the driving field from one half of the optical cycle to the next. This quantity can be straightforwardly extracted by comparing the spectrum of the signal electric field ${E_s}$ and the Fourier transform of equation (2), both of which are accessible experimentally. Following a trivial Fourier analysis, the electric field of the signal pulse can be reconstructed. A more detailed description of the reconstruction method can be found in section II of the supplementary data. The excellent agreement between the input and the reconstructed signal electric field as shown in Fig. 1(b) verifies the validity of the current method. Considering the attosecond slits are separated by half a cycle of the signal pulse, equation (2) indicates that the energy shift tends to be more profound for shorter pulses and diminishes as the pulse approaches a monochromatic field. Therefore, the current method is advantageous for characterizing shorter pulses. For longer pulses, the reduced energy shift will require a spectrometer with higher spectral resolution. This can also be understood from the perspective of frequency domain. The electric field of the signal can be retrieved if the destructive interference minimum (as shown in Fig. 3(b)) is measurable. Shorter signal pulses are beneficial as they have wider spectral bandwidth.

## WAVEFORM SAMPLING OF AN OPTICAL PULSE

**Figure 2.**
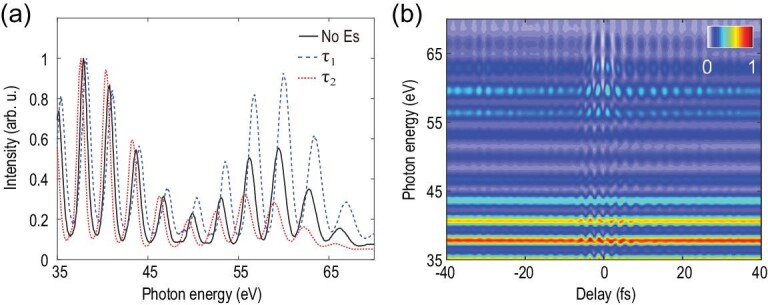
Experimental confirmation of the attosecond interferometry. (a) Measured high harmonic spectra without (black solid line) and with (dashed lines) the perturbing signal pulse. The harmonics experience maximal blue (blue dashed line) and red (red dotted line) shift at different delays due to interference between consecutive attosecond slits. (b) Normalized two dimensional spectrogram of the high order harmonic radiation. The measured interference pattern resembles the theoretical prediction.

**Figure 3.**
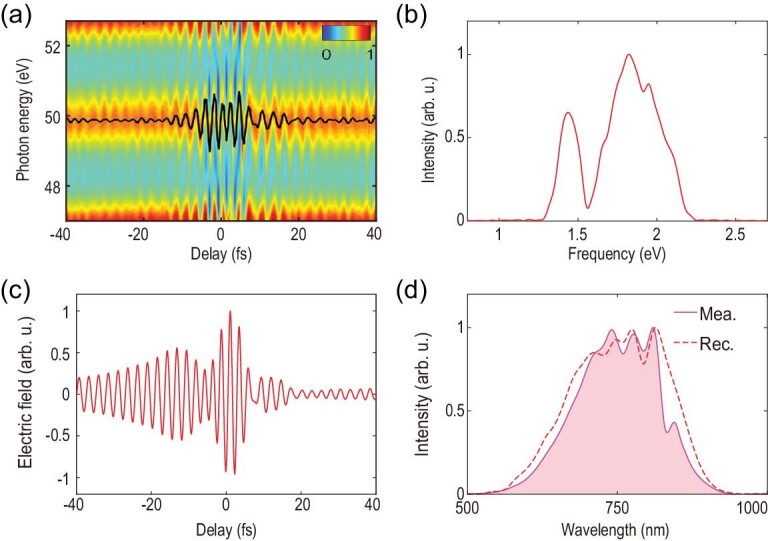
Reconstruction of the waveform of a linearly polarized signal pulse. (a) Enlarged spectrogram of the high order harmonic radiation in Fig. 2(b). The black solid line indicates the centroid of the harmonic near 50 eV. (b) Power spectrum of the black solid line of (a). (c) Retrieved electric field of the signal pulse following the method introduced in section II of the supplementary material. (d) The reconstructed (red dashed line) and measured (pink solid line) spectrum of the signal.

In the experiment, a 7 fs few-cycle laser pulse spanning from 550 nm to 900 nm is used for high harmonic generation. The focusing geometry of the driver is carefully tuned to phase-match the short trajectory such that a robust attosecond pulse train is formed. Assuming the intrinsic chirp is the main cause of pulse broadening, the pulse duration of individual attosecond bursts near the plateau region (35 eV to 60 eV) is estimated to be about 370 as. A weak signal pulse with an intensity below 1% of that of the driver is picked off to perturb the harmonic generation process. Figure 2(a) shows the impact of the weak signal field on the measured high harmonic spectrum. As the signal pulse is in (out of) phase with the driver, the spectral cutoff, as well as the individual harmonic peaks, experience a blue (red) shift relative to the case in which no signal pulse is present. The constructive (destructive) interference between the signal and driver can increase (decrease) the peak intensity of the combined electric field and leads to the extension (reduction) of the spectral cutoff [33,34]. However, the shift of each harmonic peak is a clear signature of phase variation imposed in the attosecond pulse train by the signal field. The delay-dependent high harmonic spectrum is shown in Fig. 2(b) and resembles the pattern that is predicted by the quantum simulation shown in Fig. 1(a). Figure 3 illustrates the reconstruction of the signal waveform used in the experiment. A harmonic of argon located around 50 eV is chosen for analysis. Figure 3(b) shows the power spectrum of the delay-dependent energy shift. The spectrum spans from 1.3 to 2.3 eV and corresponds to the spectral range covered by the signal field. The minimum around ${\omega _d}$ ∼ 1.6 eV is due to destructive interference of the two delayed signals depicted in equation (2). From this minimum, the time interval of two consecutive attosecond slits Δ can be accurately calculated: $\Delta = \frac{\pi }{{{\omega _d}}}$. With Δ known, it is straightforward to extract the complete information of the electric field, which is illustrated in Fig. 3(c).

The above analysis is based on a double-slit configuration. In a real experiment, an intense, few-cycle driver normally opens more than two attosecond slits depending on the harmonic order. As shown in Fig. 2, the width of individual harmonics becomes progressively wider as the photon energy increases, indicating that the number of attosecond pulses increases with decreasing harmonic order. To prove the validity and generality of the reconstruction scheme, we calculated the energy shifts for different numbers of slits by applying an appropriate window function. The calculation results show that the energy shift traces are almost identical for a slit number up to five (section II of the supplementary data). This result indicates that equation (2) still holds for a few-slit configuration as, in a multi-slit experiment, the dominant contribution to the fringes is the interference between two adjacent slits. Therefore, the current scheme for waveform reconstruction applies for a few-slit interferometer and can be easily implemented by using a few-cycle driver pulse. The actual duration of the attosecond pulses can vary slightly from run to run due to different phase matching conditions for harmonic generation. However, it is the shift of the central energy rather than the width of each harmonic that is used for retrieving the electric field of the signal pulse, meaning that the current method is not sensitive to the temporal width of the attosecond slit. Note that we used a carrier envelope phase (CEP) un-stabilized laser in the experiment. The attosecond pulses are emitted at specific phases of the driving field according to the three-step model [35]. Changing the CEP does not influence the emission time of the attosecond pulses with respect to the phase of the carrier of the driver, but rather the relative intensity of the attosecond pulses. By using a robust active delay stabilization system, the signal pulse is phase-locked with the driver at high precision (root mean square (RMS) < 15 as, see section IV of the supplementary data). This locking system is essential for successful reconstruction of the oscillating carrier of the signal pulse since it is the relative phase between the attosecond slits and the perturbing signal that determines the harmonic energy shift. Assuming the CEPs are distributed over [$ - {\rm{\pi }}$, ${\rm{\pi }}$] with equal probability [36], what we reconstructed in the experiment is actually a CEP averaged waveform. Compared to the waveform retrieved from a CEP-stabilized laser, the CEP-averaged waveform contains the full carrier information of the signal such as high order dispersion, which is a very important feature of a short pulse, but with a slightly elongated envelope. This is similar to an attosecond streaking experiment using a CEP-unstabilized laser [37]. Therefore, although the current method is applicable to measure the waveform of a CEP-fixed laser pulse in principle, when a CEP-unstabilized laser is used, the current method can still precisely measure the carrier of the laser field with the envelope information averaged. Further measurements show that the material-induced dispersion of the short pulse can be accurately recovered using the current method (section III of the supplementary data). We therefore have demonstrated the reconstruction of the waveform of a linearly polarized optical pulse.

**Figure 4.**
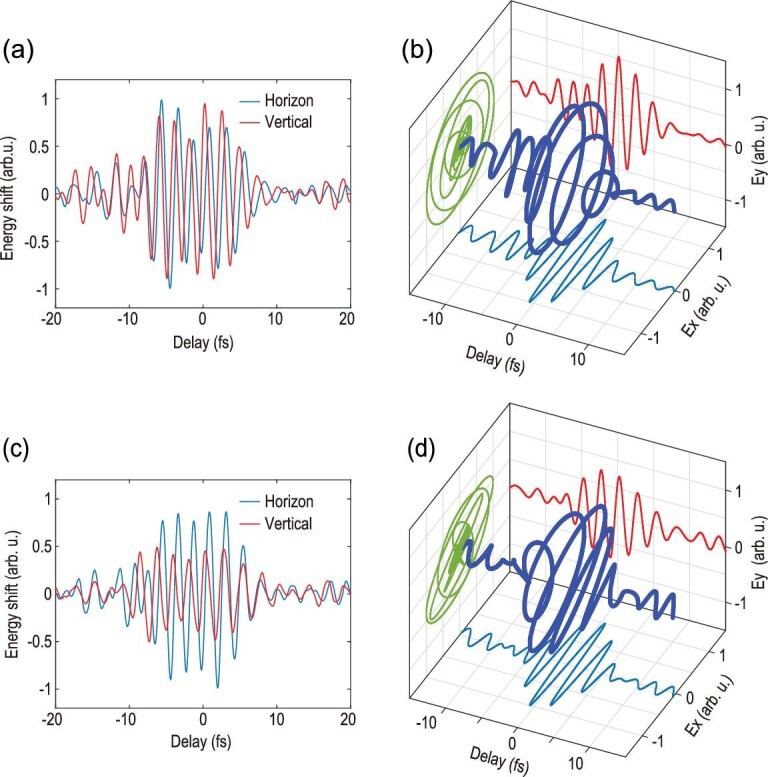
Reconstruction of the waveform of vectorial signal field. The measured delay dependent energy shifts of harmonic near 50 eV with the two polarized components parallel to the polarization direction of the driving field for a circularly polarized (a) and an elliptically polarized (c) signal pulse. The reconstructed electric field for the circularly polarized signal (b) has an ellipticity close to 1. The reconstructed electric field for the elliptically polarized signal (d) has an ellipticity of 0.65 as compared to 0.58 in theory.

This method can also be generalized for reconstructing signals with an arbitrary state of polarization as well. Since the polarization component of the signal perpendicular to the driver contributes negligibly to the phase variation of attosecond slits at the intensity used here [10], the two orthogonally polarized components of the signal pulse can be reconstructed independently by simply rotating the polarization of the signal by 90 degrees. The strong driving pulse is horizontally polarized (we define the horizontal direction as the x direction) in our experiment. A zero order quarter-wave plate followed by a zero order half-wave plate (750 nm) is inserted in the signal arm for polarization control. The ellipticity of the signal pulse is controlled by rotating the quarter-wave plate. Then, by rotating the half-wave plate, the two orthogonal polarization components of the signal field can be selected to match the polarization of the driver for diagnosis. The detailed experimental method can be found in section IV of the supplementary data. Figure 4 shows the reconstructed waveform of two elliptically polarized perturbing signals, demonstrating that our method is sensitive to the phase and amplitude ratio of the two components of the signal field and can measure signal field waveform with great accuracy. We have thus readily implemented a petahertz optical oscilloscope. Other methods, such as frequency-resolved optical gating for complete reconstruction of attosecond bursts (FROG-CRAB), also allow for the complete characterization of the waveform of an optical laser field [12]. FROG-CRAB requires the accurate detection of the photoelectron energy spectrum and the central momentum approximation is used in the reconstruction procedure. As a comparison, the current interferometric method is an all-optical waveform sampling scheme and does not require further assumptions except that the perturbing field is sufficiently weak. Therefore, it offers an alternative simple and reliable approach for electric field reconstruction.

## PROBING ABNORMAL STRUCTURE AROUND A COOPER MINIMUM

**Figure 5.**
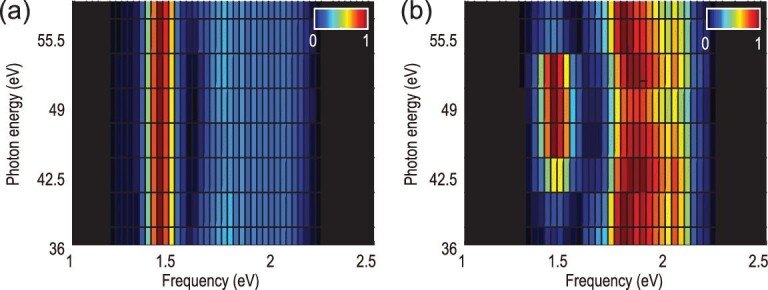
Comparing two generating targets. Fourier transform of energy shift along the delay axis for harmonics from 36 to 59 eV. Two different generating targets are used: neon (a) and argon (b). For each harmonic (horizontal axis), the spectrum has been normalized to its maxima.

Optical oscilloscopes based on high order harmonics have been reported previously using isolated attosecond pulses [10,13,14]. In the current scheme, an optical oscilloscope is realized by using an attosecond pulse train instead. The spectral resolution is crucial for performing channel-resolved measurements that are generally hindered when isolated attosecond pulses are used due to the time-energy uncertainty relationship. The intrinsic energy resolution provided in the current interferometric technique therefore implies a second application in probing structural information. The separation of the two consecutive slits Δ is trackable from the Fourier transform of the energy shift trace of a single harmonic. When multiple harmonics are considered simultaneously, an energy-resolved analysis of the temporal structure of the attosecond slits can be performed. Figure 5 illustrates the spectral analysis of the delay-dependent energy shifts for harmonics spanning from 36 to 59 eV. Two different harmonic generation targets, argon and neon, have been investigated. In the case of neon (Fig. 5(a)), the destructive interference minimum is located at around 1.6 eV and is almost independent on the harmonic order. This result is expected for high harmonic generation from targets such as neon with regular atomic structure, i.e. the phase of the recombination dipole matrix element is smooth over the range of the harmonic spectrum of interest. It reflects that the generated attosecond pulse train has a regular temporal structure where constant pulse separation is maintained over a broad range of photon energies. When the generating target is argon (see Fig. 5(b)), an abnormal shift of the destructive interference minimum is observed around a central photon energy of 50 eV over a range of more than 10 eV. This minimum is shifted towards a higher frequency and corresponds to a reduced separation between two attosecond pulses around this photon energy. The attosecond pulse separation can be calculated directly by Δ$\ = \frac{\pi }{{{\omega _d}}}$, indicating that the measurement accuracy of the position of the frequency minimum${\rm{\ }}{\omega _d}$ determines the accuracy of Δ. According to the Fourier transform property, this accuracy mainly depends on the maximal range of the delay window, which is inversely proportional to the frequency resolution when Fourier transform is performed along the delay axis. The shift of the dip shown in Fig. 5(b) indicates that the temporal separation of two successive attosecond pulses drifts from 1280 attoseconds at around 36 eV to 1200 attoseconds at around 48 eV (see section VI of the supplementary data for details). This reshaping of the attosecond pulse is likely due to the abnormal atomic structure of the generating target. It is known that a Cooper minimum (CM) exists around 50 eV in the argon harmonic spectrum and is caused by the interference between different channel contributions in the recombination step [38]. The total recombination dipole undergoes a phase jump near this minimum and alters the time structure of the radiating EUV pulses [39]. To prove that it is the Cooper minimum that leads to the abnormal shift in Fig. 5(b), we performed a model calculation based on the SFA. In the simulation, we used a dipole similar to reference [39] to mimic the interference between the s and d contributions (see supplementary data, section V). The calculation result is consistent with the discussed experimental results and thus provides evidence that this all-optical interferometry is a sensitive technique for structural determination. The current wave-front-controlled interferometer thus demonstrates the capacity to track subtle changes of the attosecond pulse separation as small as a few tens of attoseconds. Since both temporal and spectral domain information represent significant aspects of the interrogated signal, the accurate energy-resolved characterization of the attosecond slit separation provides important information necessary to grasp the dynamics around the Cooper minimum and serves as an important complement to the spectral phase measurements demonstrated in previous works [16,17].

## CONCLUSION

In summary, we present an all-optical Young's interferometer using attosecond temporal slits and explored its application for high precision measurement. High order harmonics driven by an intense few-cycle optical pulse is naturally a perfect time-energy domain few-slit interferometer. By synchronizing a delayed perturbing signal, the control of the wave-front of the temporal slits is realized, leading to an energy shift of individual harmonics. This scheme provides a two-dimensional interferogram bearing both superb time and adequate energy resolution. An intuitive analytical formula is derived to depict the perturbing field-induced energy shift. The delay-dependent shift of a single harmonic provides sufficient time resolution for attosecond sampling of a petahertz electromagnetic field. When the shifts of multiple harmonics are considered, the time separation between consecutive attosecond pulses becomes trackable in an energy resolved way. The reshaping of EUV temporal structure near a Cooper minimum in argon is revealed. As compared to previous all-optical interferometric approaches which emphasize either high temporal resolution for probing ultrafast processes [8–10], or high energy resolution for atomic phase retrieving [15–17], the current interferometry based on attosecond pulse train extends high precision measurement to a domain where both time-resolved and energy-resolved information is accessible. This wave-front controlled interferometry can potentially find significant applications in probing structural dynamics of complex molecular targets.

## METHODS

In these experiments, the 25 fs, 800 nm laser pulse from the amplifier (Legend Elite HE+ USX) is sent into a hollow-core fiber system filled with neon for spectral broadening. Pulse compression is implemented using double angled chirp mirrors (Ultrafast Innovations PC70). A 7 fs laser pulse spanning from 550 nm to 900 nm is obtained to drive high order harmonics. The static gas cell for harmonic generation is 1 mm long and filled with 30 Torr argon gas (or 100 Torr neon gas). Phase matching of the short trajectory is fulfilled by placing the gas jet 2 mm downstream of the laser focus. A small fraction of the total pulse energy is picked off to serve as a perturbing signal field using a broadband beam splitter. It is then recombined with the driver by a compact Mach-Zehnder interferometer. The relative delay between the two pulses can be fine-tuned by a piezoelectric transducer with a delay step of 300 as. The power of the driver and signal can be controlled using diaphragms. Inside the gas cell, the intensity of the driver is ∼3 × 10^14^ W cm^−2^, and the intensity ratio between the weak perturbing field and the intense driver is maintained below 1%. The high harmonic spectrum is measured with a home-built high resolution EUV spectrometer consisting of a 1200 lines/mm flat field grating (Shimadzu 30-002) and a charge coupled device (CCD) camera (Princeton Instrument PXO-400B). The harmonic spectrum at each delay is accumulated for 5000 laser shots.

We used the Lewenstein model [31] to calculate high order harmonic spectrum. The time-dependent dipole moment of an atom in a strong field is given as:

$$\begin{eqnarray*}
D(t) &=& i{\int_{0}^{\infty }{{d\tau\! \left({\frac{\pi }{{\varepsilon + i\tau /2}}} \right)}}^{3/2}}{d^*}[{P_s}(t,\tau ) \\
&&-\, A(t)]{a^*}(t) \times \exp ( - iS({P_s},t,\tau ))\\
&& \times\, E(t - \tau ) \cdot d[{P_s}(t,\tau ) - A(t - \tau )]\\
&& \times\, a(t - \tau ) + c.c.,
\end{eqnarray*}$$

where *d*(*p*) is the transition dipole matrix element between the ground state and a continuum state with momentum *p*; *E*(*t*) and *A*(*t*) represent the electric field and the associated vector potential of the combined driving and signal field; *ϵ* is a positive regularization constant; and *P_S_* and *S*(*P*, *t*, *τ*) are the canonical momentum and quasi-classical action of the continuum electron at the saddle point, respectively. Ground state depletion is included by introducing the ground state amplitude $a(t) = \exp [ { - \frac{1}{2}\int_{{ - \infty }}^{t}{{w(t^{\prime})dt^{\prime}}}} ]$, where *w*(*t*) is the ionization rate calculated with the Ammosov-Delone-Krainov (ADK) model [40]. The Fourier transformation of *D*(*t*) is denoted as the induced dipole *D*(*ω*) and the HHG intensity is proportional to *ω*^4^|*D*(*ω*)|^2^.

## Supplementary Material

nwaa211_Supplemental_File
