# Supplementary material for: All-optical attosecond time domain interferometry
Source: Natl Sci Rev. 2020 Sep 4;8(10):nwaa211. doi: 10.1093/nsr/nwaa211 (PMC8566176; doi:10.1093/nsr/nwaa211)
Supplement: nwaa211_Supplemental_File [file nwaa211_supplemental_file.docx]

**All-optical attosecond time domain interferometry: supplementary information**

Zhen Yang^1^, Wei Cao^1^, Yunlong Mo^1^, Huiyao Xu^1^, Kang Mi^1^, Pengfei Lan^1^, Qingbin Zhang^1^ and Peixiang Lu^1,2,3^

*^1^Wuhan National Laboratory for Optoelectronics and School of Physics, Huazhong University of Science and Technology, Wuhan 430074, China*

*^2^Hubei Key Laboratory of Optical Information and Pattern Recognition, Wuhan Institute of Technology, Wuhan 430205, China*

*^3^CAS Center for Excellence in Ultra-intense Laser Science, Shanghai 201800, China*

We provide important information to support the feasibility of our work in this supplementary material. In section I, the analytical formula of the harmonic energy shift under the condition of two slits interfering is derived. In section II, we numerically simulate the high harmonic spectrum using the strong field approximation (SFA) model. The simulated energy shift shows good agreement with the analytical expression and proves the validity of the method. Furthermore, we verify that the derived expression in section 1 is still applicable for multi-slit configuration. In section III, we show the measured energy shift is sensitive to the amount of dispersion introduced in the signal field, indicating the feasibility of the current method for reconstructing femtosecond pulses accurately. The detailed waveform reconstruction process for elliptically polarized signal pulses is included in section IV. In section V, a simple model is utilized to analyze the reshaping of the attosecond pulse train around the Cooper minimum of Ar, which provides evidence of the utility of the current interferometry for structure determination. In section VI, the extracted time interval of different slits of Ar harmonic radiation with different precision is presented, which shows that the current interferometry characterizes the structure of HHG with high precision.

**Section I: Derivation of high harmonic energy shift of double-slit configuration**

When only the driving field is considered and only one short trajectory channel is opened, HHG can be described by SFA where the spectral phase can be written as [1]:

$$\begin{aligned} \phi\left( \omega\right)=-S\left( P,t_{i},t_{r} \right)+\omega t_{r}\#\left( S1 \right) \end{aligned}$$

where $P$ is canonical momentum; $t_{i},t_{r}$ are the ionization and recombination time of this channel, respectively; and $\omega$ is the angular frequency. S is the quasi-classical action defined as:

$$\begin{aligned} S\left( P,t_{i},t_{r} \right)=\int_{t_{i}}^{t_{r}} \left\{ \frac{\left[ P-A_{0}\left( t \right) \right]^{2}}{2}+Ip \right\}dt\#\left( S2 \right) \end{aligned}$$

where $A_{0}$ is the vector potential of the driving field and $Ip$ is the ionization potential of target gas. The velocity of the electron in field after tunneling can be written as:

$$\begin{aligned} V\left( t \right)=P-A_{0}\left( t \right)\#\left( S3 \right) \end{aligned}$$

When a second short trajectory for harmonic generation is introduced, the time interval between two short trajectories is T/2 with T as the optical cycle of the driving field. Considering a CW driving field, $E_{0}\left( t \right)=E_{0}sin(\omega_{0}t)$, we have:

$$E_{0}\left( t+\frac{T}{2} \right)=-E_{0}(t)$$

$$\begin{aligned} A_{0}\left( t+\frac{T}{2} \right)=-A_{0}\left( t \right)\#\left( S4 \right) \end{aligned}$$

$$P\left( t+\frac{T}{2} \right)=-P(t)$$

Then the quasi-classical action for the second trajectory is:

$$\begin{aligned} S\left( P,t_{r}+\frac{T}{2},t_{i}+\frac{T}{2} \right)=S\left( P,t_{r},t_{i} \right)\#\left( S5 \right) \end{aligned}$$

When the envelope of the driving field is considered, we have:

$$\begin{aligned} S\left( P,t_{r}+\frac{T}{2},t_{i}+\frac{T}{2} \right)=S\left( P,t_{r},t_{i} \right)+\delta\#\left( S6 \right) \end{aligned}$$

with $\delta$ accounting for the non-adiabatic condition [2]. Therefore, the phase difference of the two attosecond slits is:

$$\begin{aligned} \Delta\phi\left( \omega\right)=\frac{\omega T}{2}+\pi-\delta\#\left( S7 \right) \end{aligned}$$

A phase $\pi$ is introduced to account for the sign flip of the electric field that drives the two consecutive attosecond pulses (slits). When a perturbing signal field is introduced, the phase shift of the first slit has been presented in [3] as:

$$\begin{aligned} \sigma_{1}\left( \tau^{'} \right)=-\frac{1}{72}E_{0}\omega_{0}t_{d}^{4}E_{s}\left( \tau^{'}+\xi\right)\#\left( S8 \right) \end{aligned}$$

where $t_{d}$ is the excursion time of the electron, $\xi=2t_{r}/5$ is a constant time shift [3], and $\tau^{'}$ is the delay between the driving and the signal field. A similar derivation can be applied to the second slit：

$$\begin{aligned} \sigma_{2}\left( \tau^{'} \right)=\frac{1}{72}{\alpha E}_{0}\omega_{0}t_{d}^{4}E_{s}\left( \tau^{'}+\xi+\Delta\right)\#\left( S9 \right) \end{aligned}$$

Here, we set $\tau=\tau^{'}+\xi$ as the relative delay for convenience. $\Delta$ is the time interval between two attosecond slits. The phase shifts of two slits are opposite as the vectors of driving field are opposite between the two slits. The coefficient $\alpha$ is introduced to consider the intensity variation of the driving field within an optical cycle. Consequently, the coefficient $\alpha$ represents the intensity ratio of driving field at two consecutive half cycles. Finally, the total phase difference of the two slits can be written as:

$$\Delta\Phi\left( \omega,\tau\right)=\sigma_{2}\left( \tau^{'} \right)-\sigma_{1}\left( \tau^{'} \right)+\Delta\phi\left( \omega\right)$$

$$\begin{aligned} =\frac{E_{0}\omega_{0}t_{d}^{4}}{72}\left( {\alpha E}_{s}\left( \tau+\Delta\right)+E_{s}\left( \tau\right) \right)+\frac{\omega T}{2}+\pi-\delta\#\left( S10 \right) \end{aligned}$$

When $\Delta\Phi\left( \omega,\tau\right)=2m\pi$ with $m$ as an integer, two slits interfere constructively. We have:

$$\begin{aligned} \frac{E_{0}\omega_{0}t_{d}^{4}}{72}\left( \alpha E_{s}\left( \tau+\Delta\right)+E_{s}\left( \tau\right) \right)+\frac{\omega T}{2}+\pi-\delta=2m\pi\#\left( S11 \right) \end{aligned}$$

Hence, the high order harmonics will peak at:

$$\begin{aligned} \omega=\left( 2m-1 \right)\omega_{0}+\frac{2\delta}{T}-\frac{E_{0}\omega_{0}t_{d}^{4}}{36T}\left( \alpha E_{s}\left( \tau+\Delta\right)+E_{s}\left( \tau\right) \right)\#\left( S12 \right) \end{aligned}$$

both $m$ and $\delta$ are constants and independent with the signal field. Therefore, the expression of energy shift caused by the signal field is:

$$\begin{aligned} \sigma\left( \tau\right)=-\frac{E_{0}\omega_{0}t_{d}^{4}}{36T}\left( \alpha E_{s}\left( \tau+\Delta\right)+E_{s}\left( \tau\right) \right)\#\left( S13 \right) \end{aligned}$$

**Section II: The reconstruction of the signal field and the effect of multi-slit configuration**

We performed the numerical simulation of HHG using SFA to validate the expression we derived in section I. An intense (2×10^14^ W/cm^2^) three-cycle 800nm driving field interacts with an Ar target for high harmonic generation (HHG). A weak (1×10^12^ W/cm^2^) two-cycle 800nm signal field is introduced to perturb HHG. Figure 1(a) shows the two-dimensional spectrogram of the high order harmonic radiation by scanning the delay between the two fields. In figure 1(b), we compared the energy shift simulated from SFA with that from Eq. (S13). They show good agreement when the non-[adiabatic](javascript:;) [condition](javascript:;) is considered. The small discrepancy is caused by the higher order term from Eq. (S2) ignored in the derivation [3]. Figure 1(c) shows the Fourier transform of a delay-dependent energy shift simulated by SFA near 42 eV. A dip near $\omega_{d}=1.55eV$ is due to destructive interference of the two delayed signals depicted in equation (S13) and is directly related to the time interval between the two attosecond slits $\Delta=\frac{\pi}{\omega_{d}}$. With $\Delta$ known, it is straightforward to extract the complete information of the electric field E_s_: $E_{s}\left( \omega\right)\propto\frac{\sigma\left( \omega\right)}{\left( 1+\alpha e^{i\Delta\omega} \right)}$, where $\alpha$ is adjusted to keep the spectrum of the reconstructed field similar to that of the original signal field and is in general close to 1. An inverse Fourier transform gives the time domain electric field of the signal pulse as is shown in figure 1(d). It shows that the reconstructed and original signal field are in excellent agreement, indicating the feasibility of our method. To expand the application, we increase the number of slits by adjusting the window function, which is applied for selecting the number of short trajectories in SFA. We have compared the energy shifts simulated by SFA where the multi-slit condition is considered and they appear to be almost identical (figure. 2), indicating Eq. (S13) is still feasible for multiple slits. This can be easily achieved when an ultrashort driving pulse is used for high harmonic generation.


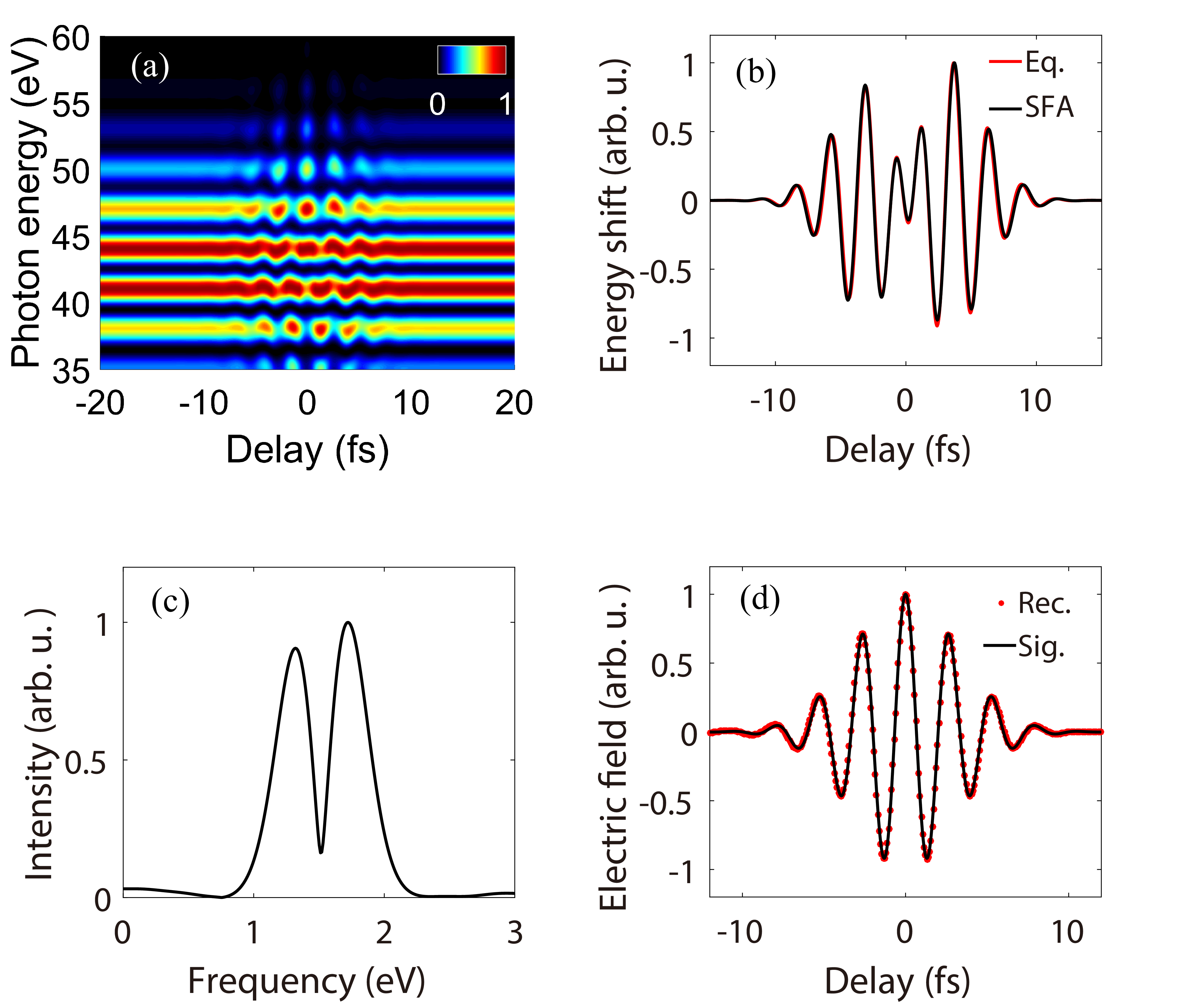


**Figure 1** (a) Two dimensional spectrogram of the high order harmonic radiation simulated by SFA. (b) The normalized results of an energy shift extracted from (a) near 42 eV (black curve) and calculated using Eq. (S13)(red curve). (c) The Fourier transform of the delay dependent energy shift in (b). (d) The reconstructed field (red dot curve) and the original signal field (black curve).


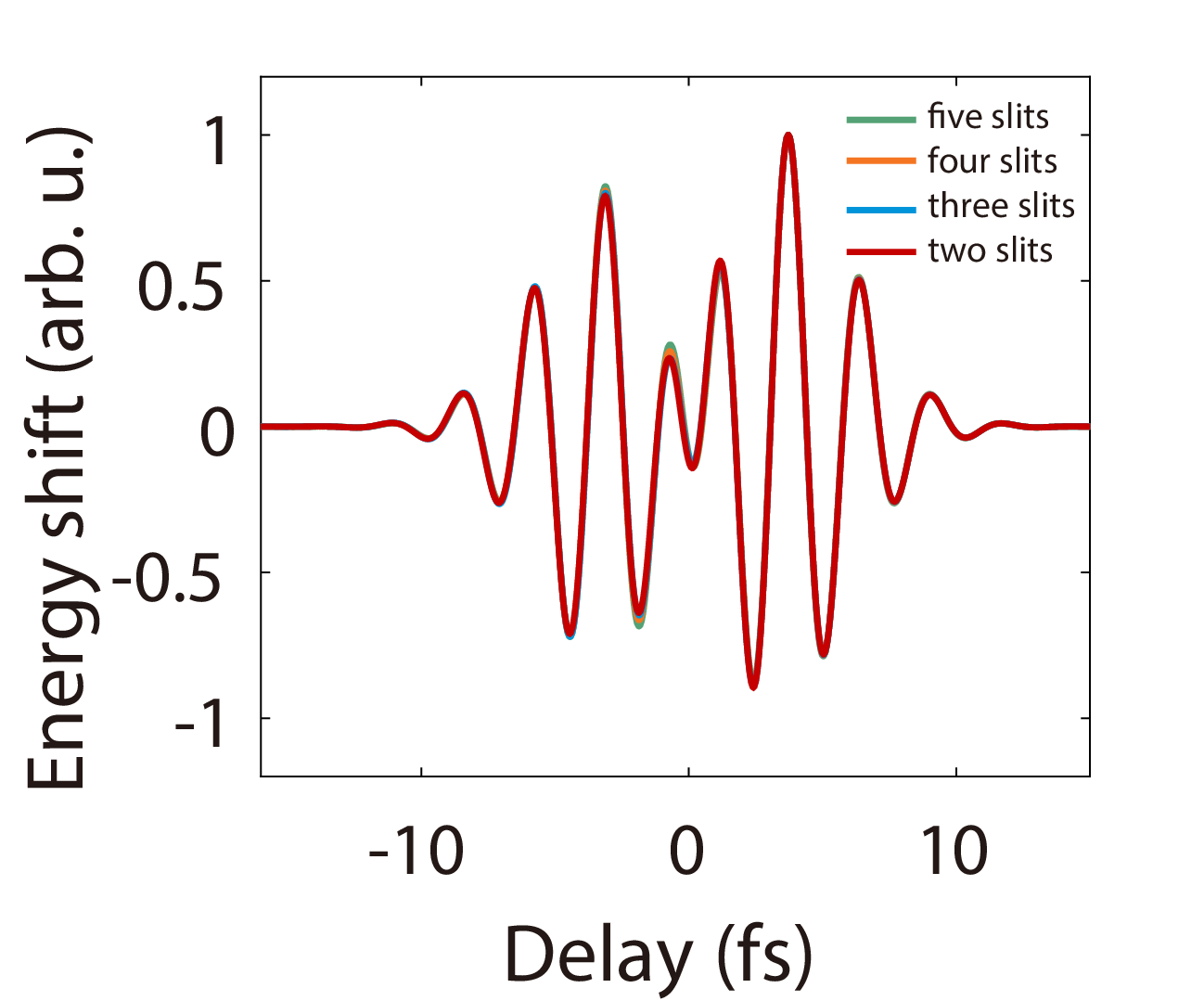


**Figure 2** The energy shift of the 27^th^ harmonic (near 42 eV) for different numbers of attosecond slits. The parameters are the same as Figure 1.

**Section III: Experimental results for chirped signal pulses**

In the experiment, we have also altered the waveform of the signal field by introducing dispersion materials to test its generality. The delay-dependent centroid of the argon harmonic near 38 eV for different thickness of fused silica inserted in the signal arm is inspected. The time-frequency analysis of the delay dependent energy shift is shown in figure 3. The position of the dip around 1.55 eV is again due to destructive interference as mentioned in section II. As the thickness of the fused silica increases, a progressive tilt of the spectrogram is observed, indicating that the signal field is gaining a positive group delay dispersion. This result implies that our scheme is sensitive enough to capture the carrier of the signal pulse.


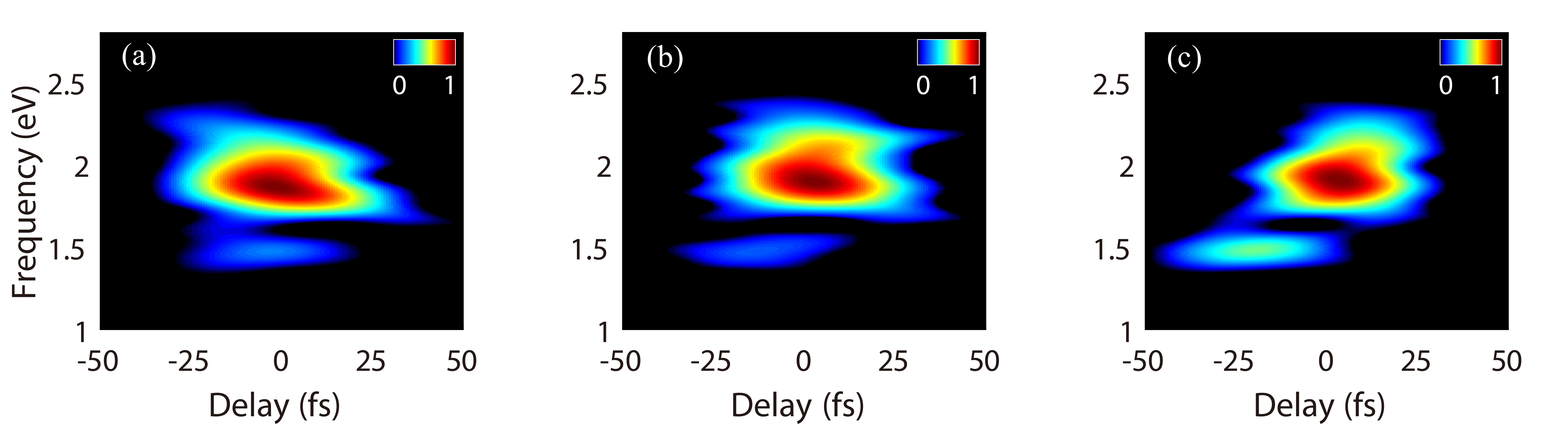


**Figure 3** The time-frequency analysis of the measured energy shift of harmonic near 38 eV after inserting 0mm (a), 0.3mm (b) and 0.5mm (c) of fused silica in the signal arm.

**Section IV: The reconstruction of elliptically polarized signal pulse by attosecond few-slit interferometry**

In this section, we introduce the detailed process of measuring vectorial optical fields. In general, a vectorial laser field can be divided into two perpendicularly polarized components. By measuring the two components independently, the total vectorial field can be reconstructed. In the present of a vectorial signal field, the total phase difference of the two slits can be written as:

$$\Delta\phi\left( \omega\right)=S\left( P,t_{i}+\frac{T}{2},t_{r}+\frac{T}{2},\tau\right)-S\left( P,t_{i},t_{r},\tau\right)+\frac{\omega T}{2}+\delta$$

$$\begin{aligned} \approx\int_{t_{i}}^{t_{r}} \left\{ \left[ P-A_{d}\left( t+\frac{T}{2} \right) \right]A_{s}\left( t+\frac{T}{2}+\tau\right) \right\}dt- \\ \int_{t_{i}}^{t_{r}} \left\{ \left[ P-A_{d}\left( t \right) \right]A_{s}\left( t+\tau\right) \right\}dt+\frac{\omega T}{2}+\pi+\delta\#S\left( 14 \right) \end{aligned}$$

Where $A_{d}$, $A_{s}$ and $P$ is the vector potential of driving field, the vector potential of signal field, and canonical momentum almost equal to $P_{0}$ ($P_{0}$ is the canonical momentum parallel to $A_{d}$ when only driving field exists) when the signal field can be regarded as a perturbation, respectively. When the driving field is linearly polarized, only the component of signal field polarized parallel to the driving field contribute to the perturbation of HHG [4, 5]. Thus, the component parallel to the driver polarization can be retrieved by the reconstruction scheme mentioned in section II. Thus, the two components of the signal pulse can be measured by rotating the polarization of the signal field or driving field by ${90}^{^{\circ}}$. We choose the latter in our simulation for convenience. Fig. 4 shows the results simulated by SFA with the same conditions mentioned in section II except that the signal field is circularly polarized instead. Figure 4(a) and (c) show the two-dimensional spectrograms of HHG perturbed by circularly polarized signal field when the polarization direction of driving field is along the horizontal (x) and vertical (y) direction, respectively. Fig. 4(b) and (d) show the reconstructed horizontal and vertical components of signal field from the energy shift extracted from (a) and (c) near 42 eV. It should be noticed that the reconstructed signal field has been normalized to the x component. Figure 4(d) indicates that the reconstructed circularly polarized signal field is consistent with the original one.


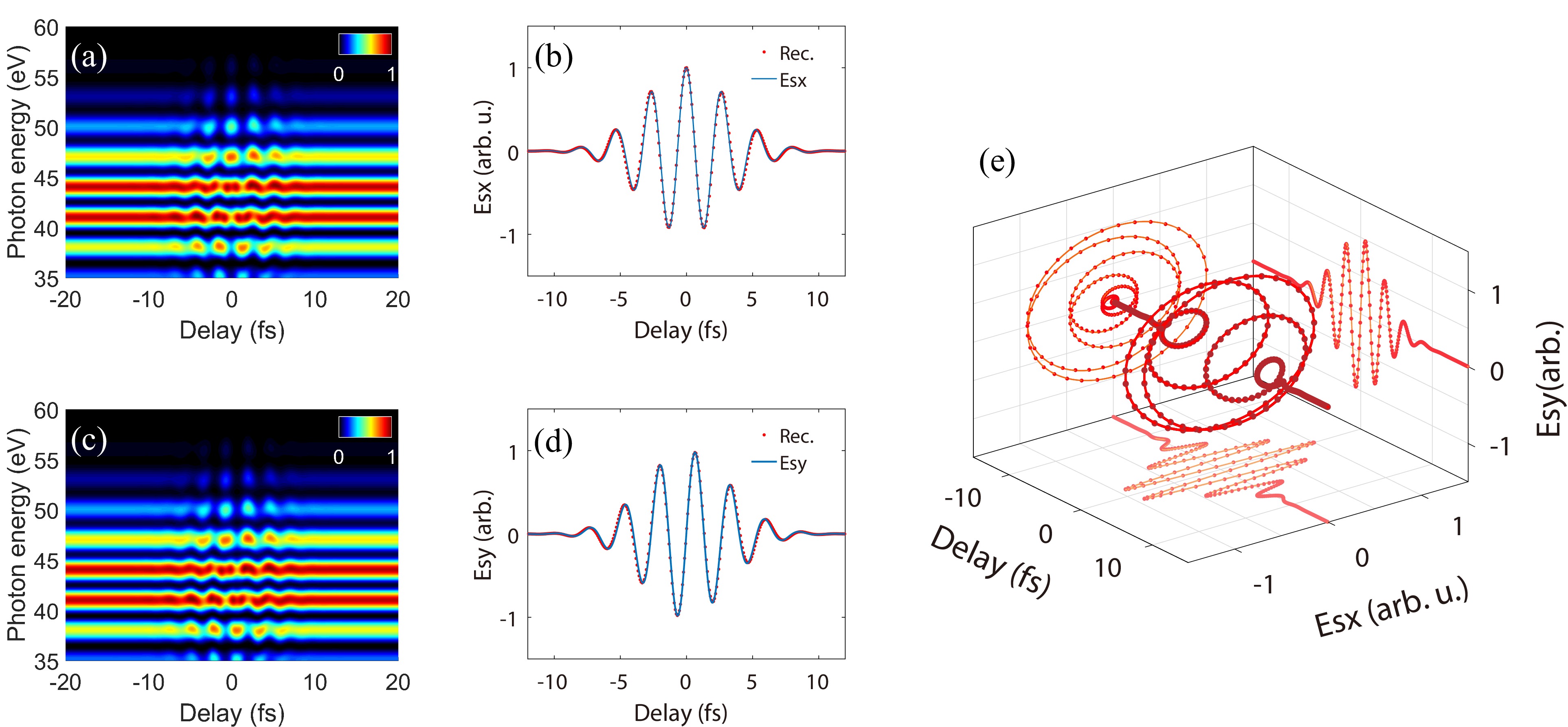


**Figure 4** (a) Calculated high order harmonic spectrum perturbed by a circularly polarized signal field with the driver polarized along x direction. (b) The reconstructed (red dots) and the original (blue solid line) x component of the signal field using harmonic near 42 eV. (c) Calculated high order harmonic spectrum perturbed by a circularly polarized signal field with the driver polarized along y direction. (d) The reconstructed (red dots) and the original (blue solid line) y component of the signal field using harmonic near 42 eV. (e) The reconstructed (orange dots) and the original (red solid line) waveform of the circularly polarized signal field.

In the experiment we rotate the signal field by ${90}^{^{\circ}}$ while keeping the polarization of the driver unchanged and perform two independent measurements for reconstructing the waveform of elliptically polarized signal pulses. Notably, the delay axis should be kept the same in the two independent experiments. To achieve this purpose, we set up an active delay stabilization system similar to [6]. Our apparatus is a Mach-Zehnder interferometer configuration (figure 5). A weak 532 nm continuous wave (CW) green laser is co-propagating with the infrared field through both arms. The spatial interference pattern of the green laser is recorded by a CCD camera. The relative delay between the two arms were determined by the interference fringes using Fourier-transform interferometry [7]. Home-built labview program was used to extract relative delay shifts and generate an error signal used to control the piezoelectric translation stage. The stabilization system was able to compensate the slow delay drifts below ~20Hz. The interferometer can be locked to within 15 as RMS (the inset of figure 5) over the entire measurement. Meanwhile, the extracted relative delay also provides a reliable reference to determine a common delay axis for two independent measurements.

The strong driving pulse is horizontally polarized (we define the horizontal direction as the x direction) in our experiment. A zero order quarter-wave plate and a zero order half-wave plate (750nm) are inserted in the signal arm for polarization control. The optical axis of both wave plates are horizontal such that both the diving and the signal pulse are horizontally polarized. A circularly or elliptically polarized signal can be generated by rotating the quarter-wave quartz plate by a certain angle. Then, by rotating the half-wave quartz plate, the two orthogonal polarization components of the signal field can be selected to match the polarization of the driver for diagnosis. It should be noticed that rotating the half-wave plate will induce a drift in the interference pattern of green laser as well because of the refractive index change of the wave plates for green light. We have taken this effect into account in order to determine an accurate common delay axis for the two independent measurements. The corrected two-dimensional spectrogram of HHG perturbed by the circularly (a1, a2) and elliptically polarized signal fields (b1, b2) are shown in Figure. 6.

It is shown that the energy shift induced by the vertical component is comparable to that of the horizontal component for circularly polarized signal pulses. In contract, the energy shift induced by the vertical component is weaker than that of the horizontal component for elliptically polarization signal pulses (see figure 5 (c) in main paper). Thus, our method is sensitive to the phase and amplitude ratio of the two components and can measure signal field waveforms with great accuracy. The reconstructed elliptically polarized signal fields are shown in figure 4 of the main paper.


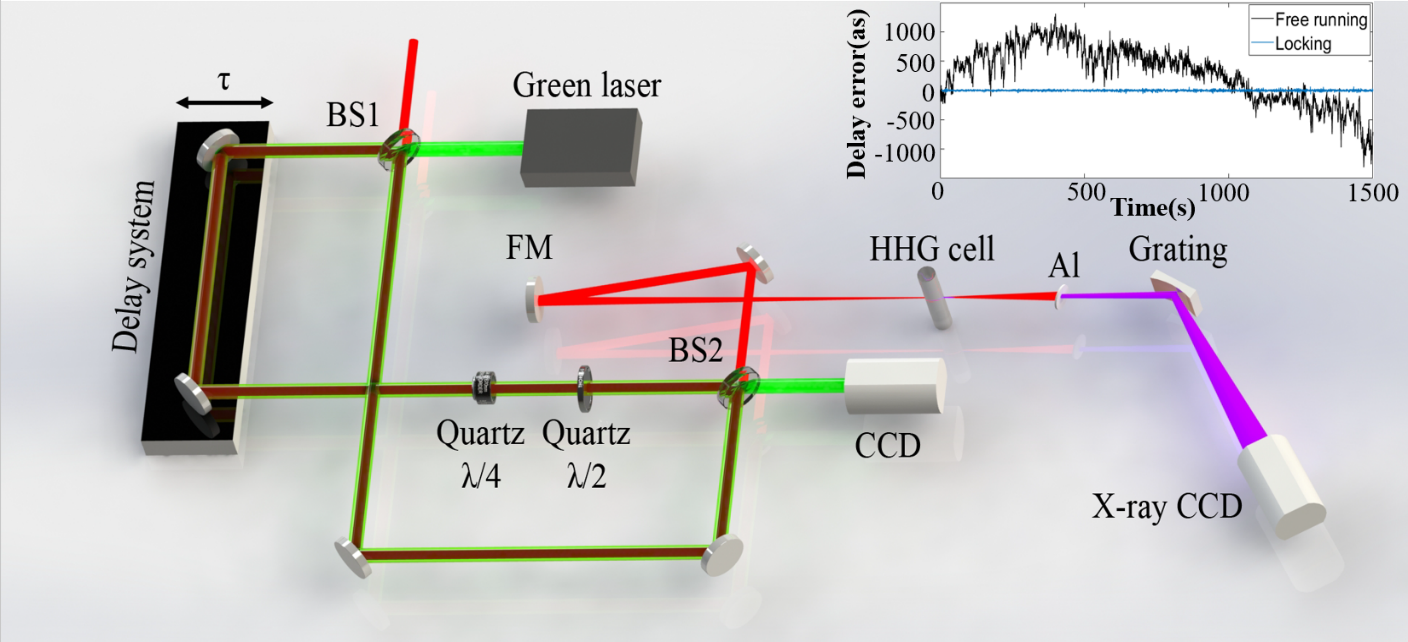


**Figure 5** Experimental set-up. BS: beam splitter, FM: focusing mirror. Al: aluminum filter for blocking the remained infrared light. The inset shows the relative delay jittering between the two arms with (blue) and without (black) the locking system. It shows that the Mach-Zehnder interferometer can be locked to within 15*as* RMS.


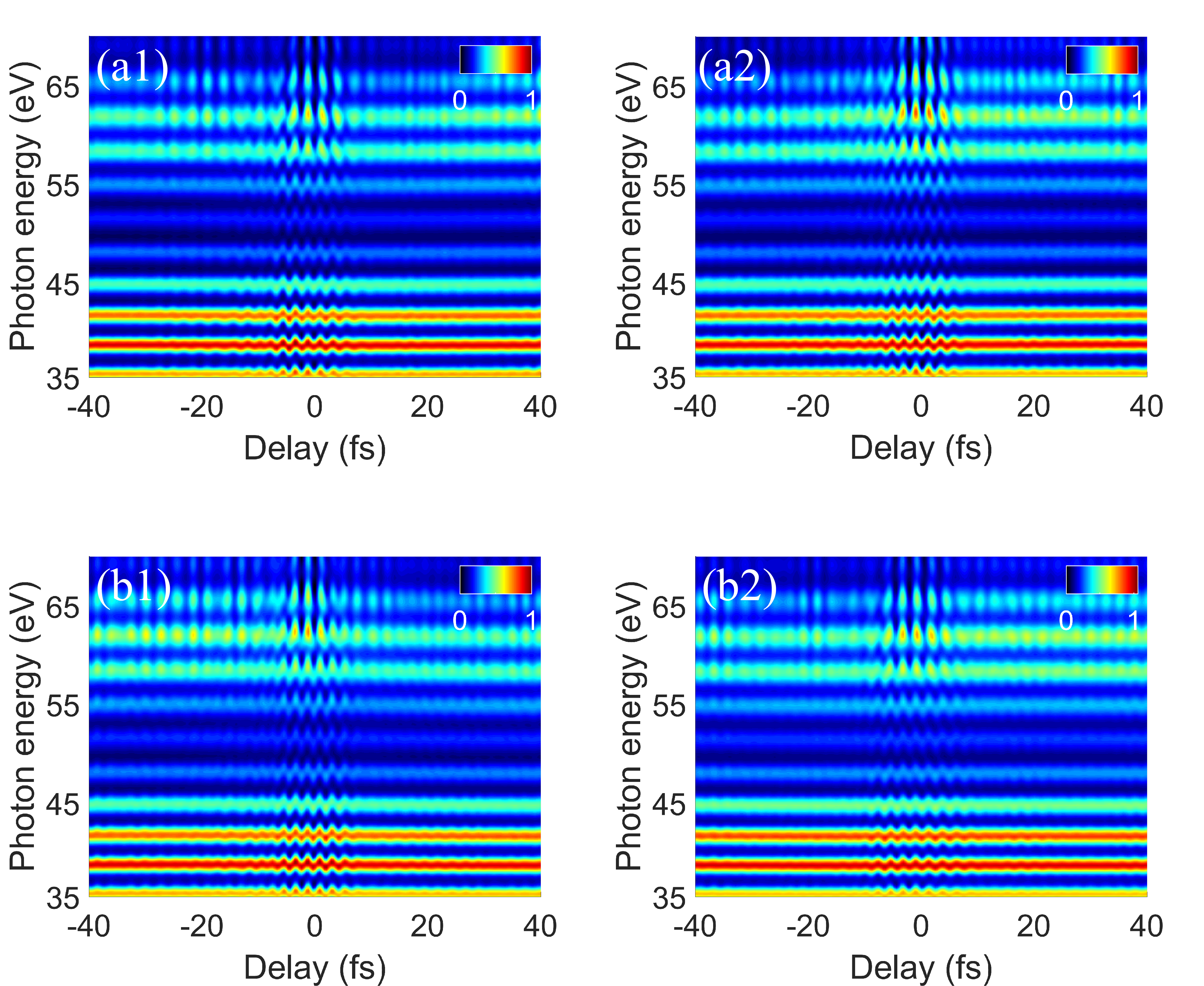


**Figure 6** The measured high order harmonic spectra perturbed by the horizontal(a1) and vertical(a2) component of a circularly polarized signal field, and by the horizontal(b1) and vertical(b2) component of an elliptically polarized signal field. For circularly polarized light, the quarter wave plate is rotated by ${45}^{^{\circ}}$. For elliptically polarized light, the quarter wave plate is rotated by ${30}^{^{\circ}}$(ellipticity equals to 0.58 in theory).

**Section V: Model calculation of the reshaping of attosecond pulse train around the Cooper minimum of Ar**

In general, the time interval of two consecutive EUV pulses is close to T/2, assuming a smooth phase distribution over the harmonic spectrum, i.e. the recombination dipole matrix element has a smooth behavior. However, when a phase jump appears in the recombination dipole matrix element, as is shown around the Cooper minimum (CM) of argon, it will alter the shape of the emitted attosecond pulses [7]. Thanks to the energy resolution provided by the current interferometric technique, the separation of the two slits can be inspected in an energy-resolved manner around the Cooper minimum. Any subtle variation in the time interval of the attosecond pulse train can be sensitively captured by interrogating the destructive interference minimum of the power spectrum of the harmonic energy shift trace, thus offering a way to study the structural features of the atomic or molecular targets. To verify this, a simple model calculation is presented here. In the simulation, both the driving field (3×10^14^ W/cm^2^) and the signal field(2×10^12^ W/cm^2^) are 800nm with a pulse duration of two optical cycles . The atomic structure is included in the calculation by using different dipole matrix elements in the SFA model. To mimic HHG in neon, we used the hydrogen-like transition dipole: ${d\left( p \right)}_{H}=\left( \frac{2^{\frac{7}{2}}\alpha^{\frac{5}{4}}}{\pi} \right)\frac{\text{p}}{\left( \text{p}^{2}+\alpha\right)^{3}}$ with p standing for the canonical momentum. In the case of argon where a Cooper minimum exists around 51 eV, we used the transition dipole in the form similar to reference[7]: $d\left( \omega\right)={d\left( \omega\right)}_{H}\left[ 1+\frac{\omega_{C}-\omega}{\Delta\omega}e^{i\xi(\omega)} \right]$ with $\omega_{C}=45.6eV$, $\Delta\omega=6.3eV$, $\xi(\omega)$=1.4 rad. $\omega$ represents the photon energy and is related to p as: $\frac{h\omega}{2\pi}=\frac{\text{ }\text{p}^{\text{2}}}{2}+I_{p}$ with$I_{p}$ the ionization potential of the generating atom. This transition dipole has a phase jump of 2.2 rad across a range of 20 eV centered at 51 eV and creates a minimum in the harmonic spectrum yield around 51 eV that resembles the main features of argon HHG. We applied a window function to select the short trajectories in the final dipole moment. Harmonic spectra for atoms without abnormal atomic structure is shown in figure 7(b). The Fourier analysis of the energy shifts of individual harmonics (figure 7(d)) gives a common minimum position around 1.55 eV that is independent on the harmonic order. Harmonic spectra for atoms with an abnormal atomic structure is shown in figure 7(a), and a minimum around 51 eV in the harmonic yield is clearly observed and indicates the CM. Figure 7(c) shows the power spectrum of the energy shift of individual harmonics. The minimum position in the power spectrum shifts towards the higher frequency components around the CM, which is consistent with the experimental results as shown in the main text. Therefore, this energy resolving capability of the current interferometry allows for the probing of abnormal atomic structure.


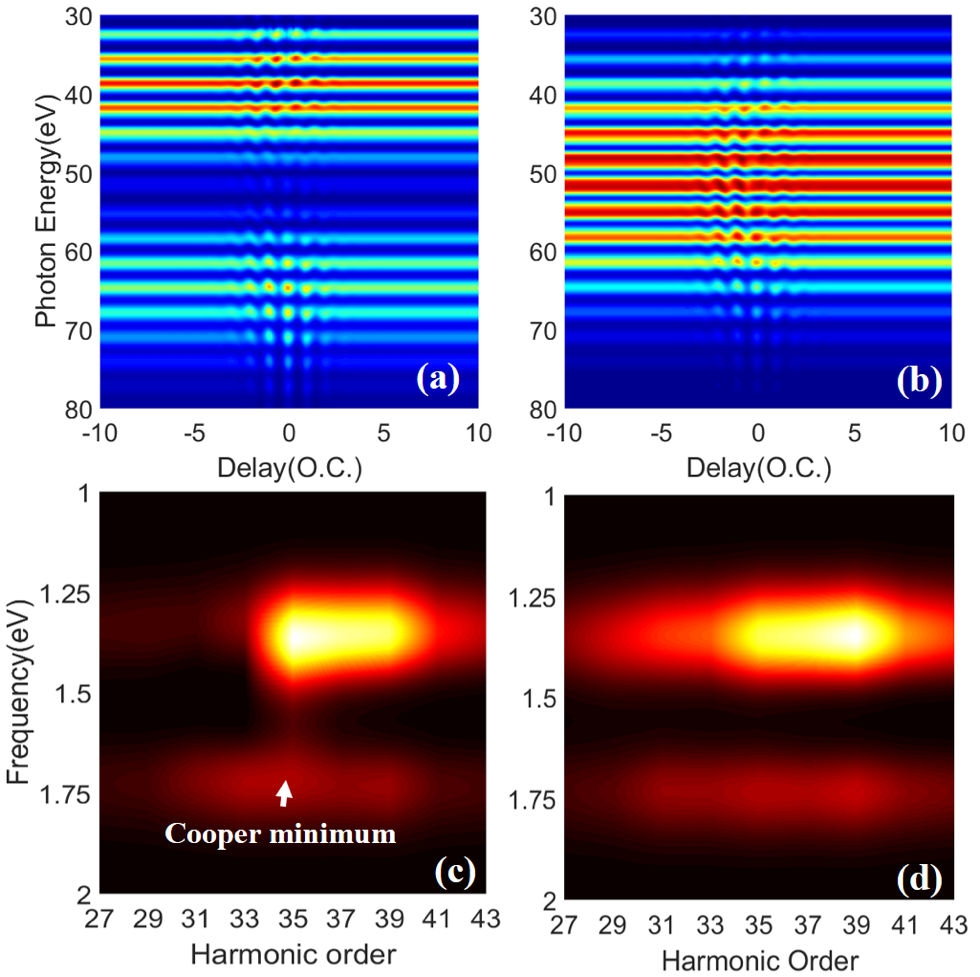


**Figure 7** Model calculation of high order harmonic spectra perturbed by a signal pulse for two different bound-free transition dipole structures. In (a), the bound-free transition dipole undergoes a phase jump near 51 eV (Cooper minimum). (c) The Fourier analysis of the delay dependent energy shift for harmonics in (a) from the 27^th^ to the 43^rd^ order. In (b), the bound-free transition dipole has a flat phase. (d) The Fourier analysis of the delay dependent energy shift for harmonics in (b) from the 27^th^ to the 43^rd^ order.

**Section VI: The extraction of time interval of attosecond slits around a Cooper minimum**

In general, the time interval of two consecutive EUV pulses is close to a half cycle of the driving pulse assuming a smooth phase distribution over the harmonic spectrum, i.e. the recombination dipole matrix element has a smooth behavior. However, when a phase jump appears in the recombination dipole matrix element, as is shown around the Cooper minimum of argon, it will alter the shape of the emitted attosecond pulses [7]. The energy resolution provided by the current interferometric technique allows an accurate energy-resolved evaluation of the time interval between two consecutive attosecond pulses around the Cooper minimum. As discussed in section II, the time interval $\Delta$ is can be calculated as : $\Delta=\frac{\pi}{\omega_{d}}$. The precision of the time interval depends on how accurately the dip position $\omega_{d}$ can be determined. According to the Fourier transform property, the frequency resolution is determined by the width of the maximal time window inspected. The precision of time interval is mainly determined by the maximal delay range accessible in the experiment. For a delay range of $\tau_{width}$= 400 fs. The corresponding frequency precision for determining $\omega_{d}$ is roughly $d\omega$= 10 meV. Thus, the accuracy of the evaluation of the time interval is $\delta\Delta$ = 8as. This implies that subtle variation in the time interval of the attosecond pulse train can be sensitively captured by interrogating the destructive interference minimum of the two-dimension spectrogram of the harmonic energy shift trace as long as the scanning relative delay is long enough. Figure 8 shows the time intervals extracted from the two-dimensional spectrograms of Ar with a delay range of 400fs. The temporal separation of two successive attosecond pulses drifts from 1280 as at around 36 eV to 1200 as at around 48 eV is clearly observed.


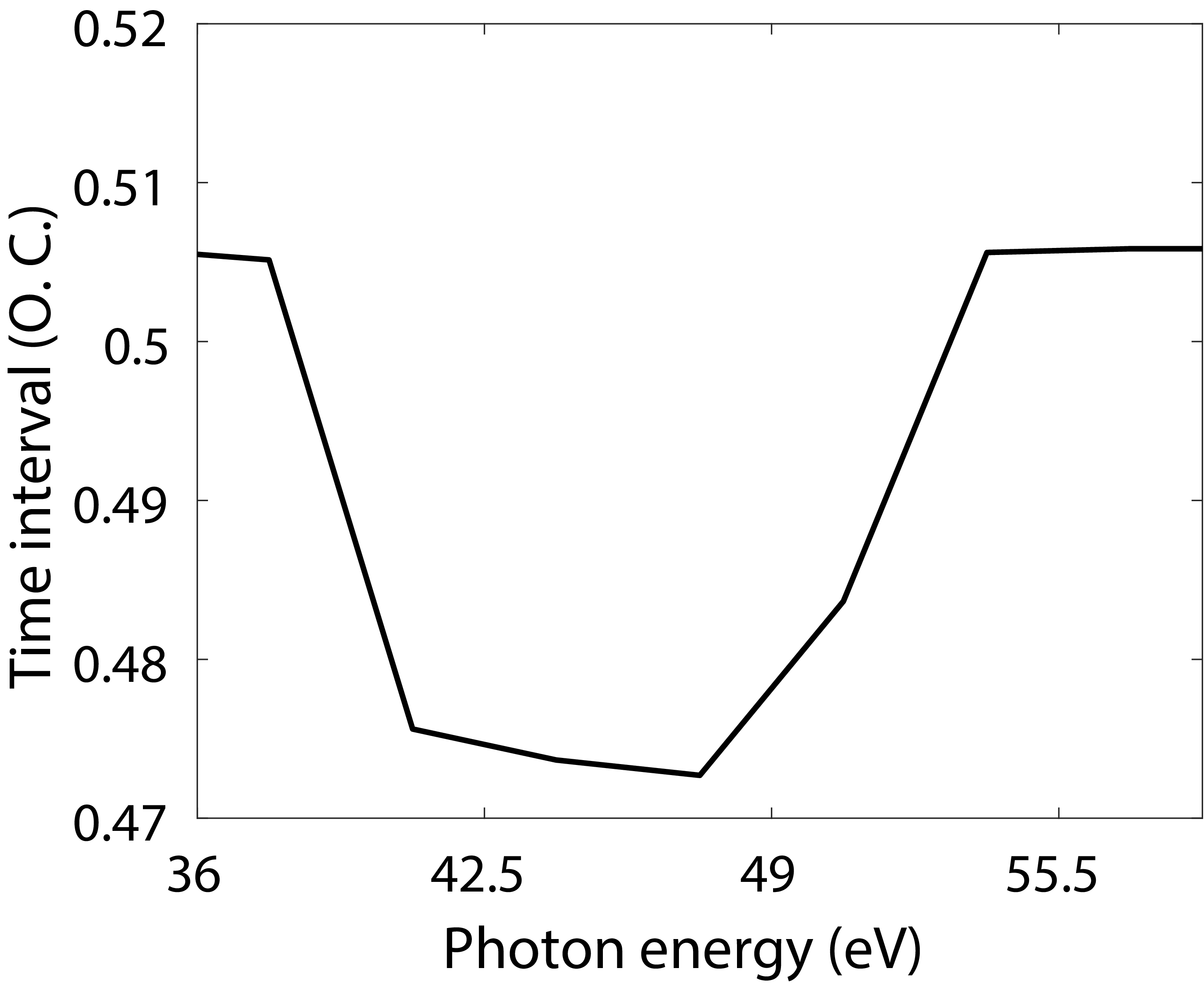


**Fig. 8** The time intervals extracted from the measured 2D spectrograms of Ar around the Cooper minimum. The delay range is equal to 400 fs. Horizontal axis indicates the time interval in unit of optical cycle of 780 nm laser field.

1. Lewenstein, M. *et al.* Theory of high-harmonic generation by low-frequency laser fields. *Phys. Rev. A,* **49**,2117 (1994).
2. Shin, H. J. *et al*. Generation of nonadiabatic blueshift of high harmonics in an intense femtosecond laser field. *Phys. Rev. Lett.* **83**, 2544 (1999).
3. Kim, K. T. *et al.* Petahertz optical oscilloscope. *Nature Photon.* **7,** 958-962 (2013).
4. Chang, Z. Single attosecond pulse and xuv supercontinuum in the high-order harmonic plateau."  *Phys. Rev. A* **70**, 043802 (2004).
5. Carpeggiani, P. *et al.* Vectorial optical field reconstruction by attosecond spatial interferometry. *Nature Photon.* **11**, 383 (2017).
6. Michael, C. et al. Delay control in attosecond pump-probe experiments. Optics express 17, 21459 (2009).
7. Lepetit,L. et al. Linear techniques of phase measurement by femtosecond spectral interferometry for applications in spectroscopy. J. Opt. Soc. Am. B 12, 2467 (1995).
8. Schoun, S. B. et al. Attosecond pulse shaping around a Cooper minimum. *Phys. Rev. Lett.* **112**, 153001 (2014).
